# *“It is challenging… oh, nobody likes it!”:* a qualitative study exploring Mozambican adolescents and young adults’ experiences with contraception

**DOI:** 10.1186/s12905-016-0326-2

**Published:** 2016-07-30

**Authors:** Rehana Capurchande, Gily Coene, Ingrid Schockaert, Manuel Macia, Herman Meulemans

**Affiliations:** 1 Department of Sociology, Eduardo Mondlane University- Maputo, Campus Universitário Principal. CP 257, Maputo, Mozambique; 2 Vrije Universiteit Brussel (Free University of Brussels), Centre for Research in Gender and Diversity, Department of Sociology, Faculty of Economic and Social Science, and Solvay Business School, Free University of Brussels, Pleinlaan 2, 1050 Brussels, Belgium; 3 Vrije Universiteit Brussel (Free University of Brussels), Department of Philosophy and Ethics, Centre for Research in Gender and Diversity, Pleinlaan 2, B-1050 Brussels, Belgium; 4 Department of Sociology, Faculty of Economic and Social Science, and Solvay Business School, Free University of Brussels, Pleinlaan 5, 1050 Brussels, Belgium; 5 Department of Sociology, Faculty of Arts and Social Sciences, Eduardo Mondlane University, Praça 25 de Junho, C. Postal 257, Maputo, Mozambique; 6 Universiteit Antwerpen (Antwerp University), Department of Sociology, Centre for Longitudinal and Life Course Studies, Sint-Jacobstraat 2, BE-2000 Antwerpen, Belgium; 7 Centre for Health Systems Research and Development (CHSR&D), University of the Free State, Bloemfontein, South Africa

**Keywords:** Adolescents/young adults’ health, Risk-taking, Barriers to contraception, Mozambique

## Abstract

**Background:**

By focusing upon formal sex education programmes, the Mozambican government has significantly enhanced the general health of adolescents and young adults. However, when it comes to contraception, little is known about how adolescents and young adults actually behave.

**Methods:**

Based upon a qualitative study in two settings in Maputo province – Ndlavela and Boane – this paper explores the knowledge and practices of contraception among adolescents and young adults. A total of four focus group discussions, 16 in-depth interviews, four informal conversations, and observations were equally divided between both study sites.

**Results:**

Discrepancies between what adolescents and young adults know and what they do quickly became evident. Ambivalent and contradictory practices concerning contraceptive use was the result. As well, young people had numerous interpretations of risk-taking when not using contraceptives. These inconsistencies are influenced by social and medical barriers such as restricted dialogue on sexuality among adolescents and young adults and their parents and peers. Additionally, ideas about indigenous contraceptives, notions of masculinity and femininity, misconceptions and fear of the side effects of contraceptives, make people of all ages wary of modern birth control. Other barriers include imposed contraceptive choice – meaning no choice, overly technical medical language used at clinics and the absence of healthcare workers more attuned to the needs of adolescents and young adults.

**Conclusions:**

Adolescents and young adults have numerous – often erroneous – opinions about contraception, leading to inconsistent use as well as vague perceptions of risk-taking. Moreover, social norms and cultural gender roles often contradict and hinder risk-avoiding behaviour. Therefore, in order to improve young people’s health, policymakers must address the reasons behind this ambivalence and inconsistency.

## Background

In Mozambique, like many other sub-Saharan Africa countries, the sexual and reproductive health (SRH) of adolescents (15–19 years-old) and young adults (20–24 years-old) remains a public health issue. This is due to several barriers such as medical and socioeconomic issues [1–3] as well as cultural values [4, 5]. These include difficulty accessing modern contraceptives; unavailability of all forms of contraceptives in deeply rural areas; shortage of health workers more suitable to attend adolescents’ needs; long waits and lack of privacy in clinics; misconceptions about certain types of contraceptives; social pressure and gender inequalities.

SRH for young people is an opportunity to provide comprehensive information about sexual health – pregnancy, sexually transmitted infections (STIs) including HIV [6]. In Mozambique, the unmet need for contraception among people of reproductive age is estimated to be 23 % [7]. The government has run campaigns promoting abstinence/delaying sexual intercourse, avoidance of risky sexual behaviour, as well as the habitual use of contraceptives [8–10]. Mozambique’s public healthcare service is the principal contraceptive supplier, servicing 77 % of modern contraception users [11]. Most family planning services are delivered through a network of state-run maternal and child health clinics. Oral contraceptives (pills), depo-provera (injections), intra-uterine devices (IUD) implants, female and male sterilisation, plus female and male condoms are offered free of charge through healthcare centres and healthcare posts [3, 8, 12].

For the purposes of this paper, contraceptives refer to several methods used by young people to avoid pregnancy, child spacing and limiting. We distinguish three types of contraception – modern, natural/traditional and indigenous contraceptives. Modern methods are based on combined oral contraceptives, progestogen-only pills, implants, progestogen-only injectables, combined injectable contraceptives, combined contraceptive patches and combined contraceptive vaginal ring, the intrauterine device, male and female condoms, vasectomy and female sterilisation [13]. Natural/traditional methods are based on fertility awareness methods, such as withdrawal and lactational amenorrhea. Indigenous contraceptives are understood within the context of traditional medicine which entails the knowledge, skills, and practices indigenous to various cultures used for the prevention or treatment of various diseases [14]. By indigenous contraceptives, we refer to contraceptive methods based on traditional local practices. This is typically a combination of herbs, amulets, charms and magical medicine, most often provided by traditional healers.

With the advent of HIV, young people’s sexual activities – considered problematic by policymakers – have become the primary focus of healthcare. The government of Mozambique treats young people’s sexual behaviour as problematic and provides services to address the problems. To illustrate, there have been concerns about teaching young people how to have safe sex, and counselling about the use of contraceptives that focus on encouragement of an earlier onset of sexual intercourse [9, 10]. However, the solutions provided for young people are not adequately addressing the context, meaning and understandings that underpin adolescent sexual behaviour. Consequently, initiatives adopted cannot work to sufficiently address the problems the Government seeks to solve.

As the literature shows, there is low acceptance of modern contraceptive use [11], including usage of SRH services [15], and the percentage of adolescents and young adults who have at least one child has increased. In 2011, the Mozambique Demographic Health Survey (DHS) showed at the national level, the rate of modern contraceptives used by female adolescents and young adults was 8 and 15 % respectively. The number of adolescents having at least one child increased as they got older. Of female adolescents aged 15 years, 11 % were mothers; 16-year-olds averaged 22 %; 39 % for 17-year-olds; 56 % for 18-year-olds, and 71 % for 19-year-old adolescents [11].

In sub-Saharan Africa (SSA) countries it is estimated that 50 % of adolescents are mothers under the age of 20 [16-18]. Indeed, early parenthood and becoming a single mother is not uncommon in many SSA countries like Ethiopia, Kenya, Malawi, Tanzania, Zimbabwe, South Africa [19] as well as Mozambique [17-18]. With early onset of childbearing and large families preferred, parenthood is seen as an essential role of family and social life towards children [20]. In particular, girls interiorize the idea that motherhood is a prerogative in their lives as women and central to female gender roles. Thus, the experience of motherhood is contextual, influenced by culture and the society within with young people live.

Studies examining the prevalence of child birth have found associations between law consistency, child marriage and adolescent childbearing [21]. Childbearing was found to be lower among women in countries with consistent laws against child marriage than among women in countries without consistent laws against such marriage [21].

Despite the window of opportunity provided by the recent family law – Law no 10/2004 regulating marriage – child marriage and early parenthood still present a social problem particularly in rural areas. Mozambique has the world’s 10^th^ highest rate of child marriage. The vast majority of child marriages are cohabitation rather than legally registered marriages but they are marriages nonetheless. These unions are formalised usually through customary procedures, such as the payment of bride price – locally termed *lobolo*– to the girl’s family [22]. According to the 2011 DHS, in rural areas, 56 % of women aged 20–24 were married by the age 18 compared with 36 % in the cities [11]. These differences may be explained by the fact that inhabitants from rural areas are more deeply influenced by traditional social and religious norms [23]. The common marital age for girls is 12 [24], and since they live in the countryside, they have less access to SRH services, compared with those living in the cities.

Overall, economic pressure and sociocultural traditions continue to drive Mozambican families to marry off their daughters at a very young age. This happens even though the girls are not old enough to take responsibilities of becoming mothers – and often leading to serious implications both for their own health and for the survival of their children [22]. Social imperatives expect girls to have a child in the early years of marriage. As well, in a patriarchal society such as Mozambique’s, a child belongs to man of the family and women are dependent upon their husbands. All these factors typically lead to early parenthood. Additionally, early sexual initiation, lack of sex education, pressure to prove adolescent fertility, child and early forced marriage, low use of contraception, and parental marital status, have been found to be contributing factors of adolescent pregnancy, child marriage and early parenthood [17]. On the other hand, the increasing presence of single parent families with women as the head of the household [25, 26] has led to vulnerabilities for young people. Being a single parent family prompts daughters to follow the same circumstances entrenching gendered vulnerability. As argued by Ramaiya et al. [16] the lack of family structure leads to the worst child outcomes; the intergenerational transmission of lower economic mobility and gender inequalities.

Motherhood is a significant aspect of life and is considered essential to women’s identity. However, when it comes to adolescents, this has been found to have adverse social, psychological, and health consequences, as well as economic costs [20, 27, 28]. In Mozambique, as in most SSA counterpart countries, children of adolescent mothers start life disadvantaged [17, 22]. Thus, the cycle of poverty and relative deprivation is perpetuated [16, 21, 29]. They experience increasing responsibility and difficulty, trying to manage the competing demands of schooling [30], and motherhood [19, 27, 31].

Adolescent childbearing is also associated with an elevated risk of serious obstetric outcomes such as obstetric fistula or death during childbirth. For the child, risks can include lower birth weight, high rate of morbidity, neonatal, and infant/child mortality [17, 28, 30]. In addition, there may be intimate partner violence [21]. In the Mozambican context, most children born to adolescent mothers are at higher risk of being malnourished and risk mortality not only in the neonatal period but up to the age 5 [22]. For this reason, young mothers must be recognised as having their own public health and intervention needs. There is a necessity to account for family circumstances and make services more youth friendly in order to reduce the cycle of vulnerability of young people in early pregnancy.

In Mozambique, between 1980 and 1990, *Population Services International* sponsored the first mass media campaign to focus on condom use. The program series targeted adolescents and young adults but made very little headway because it did not address their needs. Instead, it advocated the use of condoms only when with occasional sexual partners rather than their usual sexual partners [32]. In the end, the program had little effect other than to confuse young people about risk behaviour and the use of condoms [32, 33].

Another program called *Programa Geração Biz* (PGB), a multifaceted initiative, involved interventions in health, schools and community, aiming at improving the SRH of young people. Generally, PGB has successfully provided SRH information and services. However, it did not sufficiently address social norms contributing to adolescent SRH. As well, inadequate gender sensitivity of the PGB lead to limited effects on preventing unprotected sexual activity especially among girls [34]. Moreover, barriers related to availability, accessibility, acceptability and equity of these types of services have already been documented in the literature [15, 35].

Few studies have focused on how young Mozambicans perceive their sexual behaviour, the protective practices they adopt, [32, 36, 37], or how they act upon contraception. It is argued that several factors influence adolescent behaviour and their perception of risk. These include adolescents’ lack of accurate information, lack of sexual education at home and school [32], gender inequalities [33] and understanding of sexual pleasure [36]. There are several common misconceptions prevalent among young Mozambicans such as: “it is not necessary to use condoms in a relationship built on love” [32, 33, 37]. Another erroneous belief is that unprotected sex during the practice of *saca sena*, (one-night stand or casual sex) [36] will not result in a pregnancy or STIs. Similar risk perceptions are also found in countries neighbouring Mozambique – Malawi, Tanzania and South Africa. This reveals how sexual culture, social norms [38], sexual pleasure, permissive sexual attitudes [39], and partner dynamics affect attitudes and risk-behaviour practices leading to inconsistent use of condoms and other contraceptives [40].

Indeed, attitudes and practices concerning risk-behaviour are many and varied [41], minimal acceptance of modern contraceptives [42], and early motherhood [43] are common challenges faced by adolescents in many developing countries. A complex interaction of individual, contextual, emotional, cognitive, and behavioural factors combine with social and medical barriers to inhibit contraceptive use. These obstacles include peer influence, misconceptions about modern contraceptives [44, 45], limited communication with parents [46] and health workers, as well as social pressure including gender norms [47, 48]. As a result, adolescents engage in unsafe sexual behaviour, [49] often resulting in early pregnancies [47, 50].

Here, we explore the experiences of adolescents and young adults in relation to contraceptive use. Specifically, the focus is on knowledge, attitudes and practices connected to contraception. As well, it is of particular interest to understand perceptions around risk-behaviour, and at what point not using contraceptives is considered risky from the perspective of young people. We also delve into contraceptive use and risk-taking associated with self-concepts of risk. Lastly, social and medical barriers to contraceptive use are also explored.

This research is an attempt to understand the concept of “stock of knowledge” [51], understood as task-at-hand knowledge developed by participants in and through their everyday practices. This study starts with the assumption that adolescents and young adults have a “stock of knowledge” that might be used to make decisions about safer sex and their sexual behaviour. Stock of knowledge plays a fundamental role in how young people intuit, expect to obtain and manage pertinent information. It also involves who is to be trusted, what kind of behaviour is considered appropriate, and in what circumstances, should risk-taking be considered – or not. Thus, understanding the concept of “stock of knowledge” young people use to make decisions about their sexual behaviour may provide support for programmes or theories of sexual health behaviour. This support may be accomplished by identifying challenges, barriers, and strengths to build bridges between knowledge, practices and SRH policies for young people. All this can be achieved through identification of rational decisions and practices against risk behaviour and towards consistent contraceptive practices. Barriers, challenges and strengths, when disclosed and addressed, can lead to build programmes more attuned to adolescents needs and reduce health disparities.

This study therefore, may possible contribute to:

1. understanding the “stock of knowledge” adolescents and young adults use to make decisions (rational, spontaneous or based on habits expected on their social context) about their sexual behaviour,
2. understanding young people’s experiences using SRH services from their social context,
3. suggesting improvements to SRH services for young people in order to accomplish their needs – by reducing medical barriers and addressing social norms that obstruct young people’s health.

## Methods

This study was undertaken in Boane and Ndlavela, two sites in Maputo province, Mozambique. Boane is located in the south-west of Maputo province in a rural area. It encompasses an area of 815 km^2^ with a population of 134.000 inhabitants [24]. Ndlavela is a neighbourhood on the outskirts of Maputo City. It is located at the Infulene Administrative Post in Maputo province with a population of 57.246 [52].

This study is part of a broader research entitled *Unravelling the mosaic discourses, and practices about family planning in two settings of Maputo province, Mozambique.* The study sites were selected on the basis of contraceptive prevalence and geographical location. Both are located in Maputo province which in 2011 had the highest use of modern contraception, accounting for 32.8 % of married women [11]. As a result of the rural to city exodus, people from all over Mozambique mingle to create a cultural mix in both study sites. Predominantly, both study sites are populated by lower-class students whose parents are usually poor and less educated compared to students’ families living in the Mozambican capital. Parents in these rural areas generally work on small plots of land. Others are employed in informal activities such as selling vegetables and other food in the local market or in front of their houses.

Data were collected from February to the end of July, 2013 and were based upon in-depth interviews (IDIs), focus group discussions (FGDs), observations and informal conversations (ICs). A total of 42 participants were involved in this research. Among this number, we conducted all types of interviews. A total of 16 IDIs were held – eight in Boane and eight in Ndlavela. Each IDI lasted approximately 1 h and 30 min. Table 1 demonstrates characteristics of the participants interviewed during IDIs.

**Table 1:** Demographic background of the participants (IDIs)

IDIs	Ndavela/urban		Boane/rural			
	Ndlavela	Ndlavela	Boane headquarters	Boane headquarters	Mahubo	Mahubo
	Female	Male	Female	Male	Female	Male
Number of participants	4	4	2	2	2	2
Age range	15-23	17-23	20-22	16-23	15-21	19-24
Educational						
Grade 7–8	1	0	0	0	0	0
Grade 9–10	1	1	1	1	1	1
Grade 11–12	2	2	1	1	1	1
University	0	1	0	0	0	0
Type of Contraceptive use	Contraceptive use by female	Contraceptive use reported to be used by their female partner	Contraceptive use by female	Contraceptive use eported to be used by their female partner	Contraceptive use by female	Contraceptive use reported to be used by their female partner^a^
	Pills *n* = 1	Pills *n* = 0	Pills *n* = 0	Pills *n* = 0	Pills *n* = 0	Pills *n* = 1
	Injections = 1	Injections = 0	Injections = 1	Injections = 0	Injections = 1	Injections = 0
	Intra uterine devices *n* = 0	Intra uterine devices *n* = 0	Intra uterine devices *n* = 0	Intra uterine devices *n* = 0	Intra uterine devices *n* = 0	Intra uterine devices *n* = 0
	Condoms = 0	Condoms = 1	Condoms = 0	Condoms = 1	Condoms = 0	Condoms = 0
	Withdrawal *n* = 1	Withdrawal *n* = 1	Withdrawal *n* = 0	Withdrawal *n* = 0	Withdrawal *n* = 0	Withdrawal *n* = 0
	Indigenous = 1	Indigenous *n* = 0	Indigenous *n* = 1			Indigenous *n* = 1
		Do not know *n* = 2		Do not know *n* = 1	Not any *n* = 1	Do not know *n* = 0

^a^All condoms mentioned in the table are male condoms. None participant reported to use female condom

This study also conducted a total of four FGDs; two FGDs with females and two with male participants at both study sites. Each FGD had 8–13 participants aged 15–24 years, with a duration of approximately 1 h and 30 min.

At the beginning of our research, we decided to organise mixed gender FGDs as a pre-test to determine the best way to acquire accurate information. The first thing we noticed was that young people felt embarrassed and/or uncomfortable with talking about their sexuality in a mixed gender setting. Thus, we reverted to same-gender only participants for each discussion session.

Within each gender-specific group, we included both adolescents and young adults. This last choice was due to time and funding constraints for field work, availability of participants, as well as participants’ expectations. Most were enthusiastic about discussing contraception and safe sex with people of different ages. Table 2 reveals characteristics of the participants interviewed during FGDs.

**Table 2:** Demographic background of participants attending (FGDs)

FGDs	Ndlavela		Boane	
	Ndlavela	Ndlavela	Boane headquarters	Mahubo
	Female	Male	Male	Female
Number of participants	10	8	11	13
Age range	15–22	15–24	15–23	15–24
Educational				
Grade 7	3	2	0	0
Grade 8	5	4	6	3
Grade 9	2	2	5	4
Grade 10	0	0	0	6
Type of Contraceptive use	Contraceptive use by female	Contraceptive use reported to be used by their female partner	Contraceptive use reported to be used by their female partner	Contraceptive use by female
	Pills *n* = 2	Pills *n* = 0	Pills *n* = 1	Pills *n* = 2
	Injections = 2	Injections = 2	Injections = 1	Injections = 3
	Intra uterine devices *n* = 1	Intra uterine devices *n* = 0	Intra uterine devices *n* = 0	Intra uterine devices *n* = 0
	Condoms = 1	Condoms = 1	Condoms = 3	Condoms = 2
	Withdrawal *n* = 1	Withdrawal *n* = 1	Withdrawal *n* = 1	Withdrawal *n* = 2
	Implants *n* = 0	Indigenous = 1	Indigenous *n* = 2	Indigenous *n* = 2
	Indigenous *n* = 2	Not any *n* = 1	Not any *n* = 1	Not any *n* = 2
	Not any *n* = 1	Do not know *n* = 2	Do not know *n* = 2	Do not know *n* = 0
	Do not know *n* = 0			

A total of four ICs were held. Utilising this method was helpful in creating sociability as well as an understanding of participants’ expectations in experimenting with contraceptives [53]. This included talking with participants in an informal way, and listening to their stories. We also made a point of getting acquainted with a few of their friends and participated in some activities at schools and in the community. Talking with participants without a recorder proved to be the best way to establish intimate and trusting relationships. Data from observation and informal interviews were presented in summary with the results. Table 3 shows characteristics of the participants interviewed during ICs.

**Table 3:** Demographic background of the participants (ICs)

ICs	Ndlavela		Boane	
	Ndlavela	Ndlavela	Boane headquarters	Mahubo
	Female	Male	Male	Female
Number of participants	1	1	1	1
Age	15	22	18	21
Educational level				
	Grade 8	Grade 10	Grade 10	Grade 9
Type of Contraceptive use	Contraceptive use by female	Contraceptive use reported to be used by their female partner	Contraceptive use reported to be used by their female partner	Contraceptive use by female
	condoms	Withdrawal and condoms	Injections	Indigenous and Withdrawal

Data from IDIs, FGDs, and IC were collected by the main researcher – a female 39 years of age – who developed an open-minded environment. In some instances, common slang adopted by adolescents and young adults was used in order to gain their trust. Selection criteria were based in purposive and snowball sampling. This included: being adolescent or a young adult, a student, and in touch with school sexual education programmes.

Generally, there is quite little known about contraceptive use among Mozambican young people. In spite of this, our study only dealt with students. The rationale behind this decision was our interest in understanding what happens to those who are aware of the concept of safe sex through school programmes/activities.

We also used official documents from the Ministry of Health as a means of investigation sustaining the analysis of this study. We reviewed and coded policy documents – specifically the National Family Planning Policy [9], the National Policy for Adolescents and Youth [10], and the Health Sector Policy of 2014–2019 [8]. Here, by code, we mean a word or short phrase symbolically assigning a summative, salient, essence-capturing, and/or evocative attribute for a portion of language-based data. These documents were useful in understanding how the issue of young people’s health is framed in Mozambique.

This study starts by probing the public health approach adopted by Mozambican Government in the way young peoples’ SRH problems are framed. Adolescent and young adults’ health is seen as problematic and there are solutions to reverse the scenario. The solution includes counselling services at clinics, as well as during community and school activities. However, inadequate attention to medical barriers and socio cultural and community factors underpinning young people’s sexual health may derail these initiatives. Therefore, this study is interested to detail how young people interpret contraception and risk-taking behaviour. As well, we seek to know whether or not young people were acting in accordance with how they were represented in policy documents.

### Interview guide

The main researcher wrote the first version of the interview guide and the other authors interactively refined it. The topic content was very similar for IDIs and FGDs. Nevertheless, this study used semi-structured topics for IDIs. For FGDs, we created an interactive relationship with participants by using photos of contraceptive devices. We also encouraged the asking of questions considered controversial.

These included discussing responsibility and decision-making concerning the use of contraceptives, experiences of risk-taking behaviour, the meaning of safe sex, and the consequences of early parenthood. During the FGDs, photos of unlabelled contraceptive devices were shared. Our first question within each group was: “Have you ever seen or heard about these devices or pills”?

The interviews next proceeded with questions about prior knowledge and usage of contraceptives, experiences, feelings, and expectations about contraceptive methods. During the FDGs no participant was asked about the type of contraceptive they were currently using. Information about contraceptive type was collected in private in order to avoid embarrassment. The data was collected in this way not to disclose private issues and topics often considered taboo within the participants’ culture. At the end of FGDs session, we asked participants individually for this information. By using this strategy each participant did not have to publicly disclose the contraceptive type they used. All participants who were part of IDIs and later joined as members of FGDs were asked again about the contraceptive type they currently used in order to cross reference the information provided in the previous interview.

### Data analysis

Interviews were first transcribed, then coded, and finally translated from Portuguese to English. From empirical codes, we selected open and axial codes for the study [54]. The first part of the analytical process primarily involved ‘fracturing’ the data. The collected data were examined separately for differences and similarities. Some questions used for guiding this process were: ‘Do other participants hold similar beliefs? Is there a specific theme or concept to which this issue relates?’ This process is characteristic of the constant comparative method. By looking for similarities and asking questions, concepts that in essence were very similar, were labelled with the same name. Each concept was then defined in terms of a set of discrete properties and dimensions to add clarity and understanding [54].

The second part of the analytic process dealt with the differentiation between the concepts of open and axial codes. The first is characterised by creating open codes/coding. Open codes consisted of highlighting what participants reported as occurring more than once as well as aspects of behaviour taken for granted. Open codes encompassed knowledge, language, practices, and barriers regarding contraceptives.

Once open codes were selected, we next proceeded with axial codes/coding. Because there were a large number of codes, we found it necessary to sort them into orderly groups. With axial codes, it was possible to create themes by grouping phrases and words. These comprised the labelling of disincentives and motivations for using contraceptives as well as risk-taking behaviour. Precise questions guiding this procedure were: When it comes to contraceptives, what are adolescents/young adults really doing? What are they trying to undertake? How exactly do they act? What assumptions are they making? What did we learn from these notes? By using axial coding, we were able to denote the way in which connections are made in new ways between categories and sub-categories.

We identified specific features, such as conditions giving rise to risk behaviour and the context in which the concept is embedded. This in turn, helped to achieve precision in categories and sub-categories – such as notions about femininity and masculinity. However, open and axial coding can occur in a cycle even though they are distinct analytical procedures. Themes appearing in the results section emerged from open and axial codes.

Regarding coding, this process first followed the “member checking” process. It consisted of consulting the participants during our analysis as a way of validating the findings. Following this, double-checking and discussion ensued. Thus, the authors of this manuscript were involved in all the various stages.

It should be stated, the authors, as a collective, conceived this study. However, only the first author conducted data collection, transcribed the interviews and consulted participants during the analysis. However, all authors fully participated in the coding process and cross-referenced data. Data were analysed from August to December 2014, following a phenomenological approach. Three steps were considered in the analysis: examining commonalities, differences, and relationships [54]. A triangulation method was used to discuss our data and to derive greater assurance from the findings [55]. We cross-referenced information from FGDs, IDIs, ICs, observation and literature review on adolescent health, as well as the previously mentioned policy documents.

## Results

Findings of this study included several themes – knowledge, attitudes and practices involving contraception and risk-behaviour, responsibility for contraception, notions about masculinity and femininity in relation to contraception. Also explored were power asymmetry between young people and nurses, limited parental communication with young people, as well as misconceptions and fear of contraceptive side effects. These themes offer a possible explanation for young people’s experiences with contraception, perceptions about risk-behaviour, as well as what may stand in the way of available initiatives for improving the health of young people. Although the examples given in the text are individual voices, they generally represent the perceptions of the majority of participants. In order to protect the identity of study participants, all names used in quotes are fictitious. Additionally, no more than two indirect identifiers, such as gender, age or location were included.

### “Stock of knowledge” about contraceptives methods

In Ndlavela and Boane, adolescents and young adults obtained knowledge about contraceptives from diverse sources, such as family, acquaintances, media, healthcare facilities and community initiatives:

*I have heard about contraception through school programmes, television… at home and even with my friends. Contraception is when somebody knows about how to plan his future. It’s a method to avoid pregnancy…* [Khanysa, 15 years-old, FGD, Ndlavela].

*I heard about family planning in many places and with relatives. It is related to advices, counselling that they* [adults] *give as. To start using contraceptives, girls have to go to clinics… Condoms it is for preventing for unwanted pregnancies, HIV and STIs. I have participated in some of the programmes. There are some activities of disseminating preventive attitudes for safe sex at school that are organised for us that involve all students.(…) it is important to avoid unwanted pregnancy, so we can have more time for our studies.* [Gabriel, 16 years-old, FGD, Boane headquarters].

As demonstrated by the quotes above, young people are aware of the available services of SRH. The relevance of contraceptive use is linked to planning their future, avoiding HIV and STIs, as well as unwanted pregnancies. Thus, it is not a question of lack of services and where to get information if young people need it. Sexual health campaigns have successfully instilled the concept of safe sex and pregnancy prevention into the minds of most young people.

However, participants have elementary and interconnected knowledge and beliefs about modern, natural, and indigenous contraceptive practices. Through peer influence, participants learned about indigenous contraceptives based upon cultural beliefs. However, some differences were found between gender and age groups involving attitudes about indigenous contraceptives. Among young adult females, these herbal methods are more widely believed to be effective. The following examples reveal knowledge levels of indigenous contraceptive as well as combining modern, natural and indigenous contraceptive methods.

*There is a plant which is sold on the street… I heard of it with friends…Drinking the liquid from that plant, you can prevent pregnancy…* [Matimbe, 16 years-old, IDI, Boane headquarters].

*I combine pills and indigenous… I learnt with my friends… because of the bleeding, but also I have to carry pills on my schoolbag. My parents cannot see pills.* [Beatriz, 23 years-old, IDI, Mahubo].

*(…) the tree with big leaves, called suruma, we drink it as contraceptive…Some friends introduce the seeds of the plant into vagina and it is prejudicial…you became sterile…hum…childless… I prefer to drink it. The number of seeds you drink it is linked to the number of years you can prevent a pregnancy. It is important to combine with withdrawal….* [Zabela, 21 years-old, IDI, Ndlavela].

Participants’ choice of indigenous measures is influenced by their cultural background, community and peer influence, as well as socio-demographic characteristics and availability of resources. Misinformation about side effects linked to modern contraceptives serve as a deterrent. As well, easy access to indigenous methods is a determinant allowing for the combining of several methods of birth control.

An important issue emerged from our findings. Modern and indigenous birth control are designed for women. However, it is the male partner who chooses the type. Thus, both methods are gendered. Modern and indigenous contraceptive methods are culturally expressed as being “white/western” and “indigenous/traditional” respectively. However, both are perceived as legitimate means of birth control. Indigenous methods are observed as a process of reviving traditional/cultural forms of regulating fertility as practiced by their ancestors. In rural settings, most young adults are subject to rites of passage from childhood to adulthood. Influenced by their cultural background, most participants lack in-depth understanding of the importance of consistently using modern contraceptives – as well as the concept of risk behaviour.

Very few participants were aware of the side effects of indigenous methods – pregnancy, infertility or later needing a hysterectomy – if used incorrectly. However, the use of herbs and drugs were more frequent among participants of Boane than in Ndlavela. In both study sites, it was apparent that withdrawal, injections, pills, male condoms, indigenous, and IUDs – in preferential order – were the favoured contraceptive methods for female participants in both study areas. Most male participants preferred withdrawal and contested IUDs and injections, followed by male condoms, particularly when used with a regular partner. No female participants used female condoms due to the misconceptions concerning this method. And, although indigenous contraceptives are largely known amongst adolescents, this method is not the first choice for female participants. The ability to conceal contraceptive use from partners and parents was a contributing factor for choosing a particular method.

Concealing contraception from parents is due to the fact that talking about contraception and sexuality is still a taboo within the family. It is also viewed as proof of already having sexual intercourse. On the contrary, concealing contraception from partners occurs when the male partner rejects the idea of contraception or when there is no agreement between the partners about the type of contraceptive to be used. Disagreements between partners are most often due to misconceptions about the side effects of modern contraceptives. Thus, “stock of knowledge” is used particularly by girls to make decisions about contraceptive choice. They also decide what is expected by their partners as well as in a social context. All decisions about safe sex are an illustration of what influences their rational and spontaneous decisions when addressing contraception issues.

### Gender, communication and responsibility for contraception

There were also gender differences in the perception of modern contraceptives and risk behaviour. At both study sites, male participants had less accurate information about modern contraception and were more likely to engage in multiple risk-behaviours than females. For instance, males were more prone having unprotected sexual intercourse with more than one partner. They also reported to use condoms inconsistently and did not bother to seek out SRH services. Instead, they acted like “real men” who relied on the casual female sex partner to inform them of their HIV or STI status. Interestingly, young male adults tended to be more inclined to engage in risk-taking than male adolescents. These differences between age groups and gender were more obvious in Boane than in Ndlavela:

*Boys do not even want to talk about it* [contraceptive methods and safe sex]…*If I start he gets bored. I use to talk about it with my friends. When we are into the bedroom, because I want to avoid a quarrel, I have to accept what he wants….* [Palmira, 22 years-old, FGD, Ndlavela]*.*

*It is rare to talk about contraception. With my friends we use to talk about HIV and STI. Family planning is women’s business and it’s embarrassing for us. I think it is women’s responsibility to avoid pregnancy.* [Lino, 18 years-old, FGDs, Boane].

It was clear from our findings that gendered assumptions about who is responsible for contraception affected boys’ and girls’ discussions about contraceptives and safe sex. Conversations such as these are rare between partners and in this study, most were held in gender separated groups. Gender groups differ, not only in the way they perceive, negotiate and discuss contraception issues, but also in terms of social norms and cultural values.

Boys’ conversations about contraceptives were short and predominantly characterised with discussions about HIV and STIs. Such conversations with males were embedded in the dominant patriarchal values while female communications were linked to feminine roles. Two factors may explain these attitudes. Firstly, official sex education messages emphasise more on HIV and STIs. Secondly, within community norms, contraception is understood to be private. Therefore, women talk only with other women about such matters and such embarrassing topics are avoided by males.

The responsibility for the use of contraceptives is also strongly gendered in terms of choice and preferences. Although girls are the primary users of contraceptives and “responsible for contraceptive use”, in most instances, they do not have the power to make decisions about the type of contraceptive they will use. Therefore, many girls conceal the type of contraceptive they have chosen from their partner in order to avoid conflicts. For instance, girls indicated fear of breaking up the relationship or instigating quarrels with their partner as a consequence of insisting on using an IUD or some other disputed modern method. In such cases, not all male partners know exactly what contraceptive method their partner is using. Girls must conceal the type of contraceptives that they are using as well as assimilate “responsibility” for the use of the contraception. Thus, responsibility for contraceptive use becomes a complex phenomenon particularly for girls in circumstances where they lack the power of decision making.

Modern and indigenous contraceptives are attributed to be the girl/woman’s responsibility and withdrawal is the responsibility for males. Although condoms are male-controlled methods, when it comes to the responsibility for carrying it, this becomes a multifaceted issue. Most male partners believed that using condoms demonstrates “lack of trust when used with their regular partner”. They also rationalise that with condom use, there is a “lack of pleasure”. In the end, they leave this “responsibility” and “decision” up to their female partners. There are embedded issues contributing to the girl’s “agreement” to take on this responsibility. Due to cultural norms, most female participants appeared to believe the use of condoms and all other contraceptive methods was their responsibility. Thus, women’s ability to control contraception is assumed to arise from their biological risk of getting pregnant.

However, there were various levels of responsibility and control not only regarding condoms but also on other contraceptives, depending on the stage of the relationship and whether a girl had experienced an unplanned pregnancy or not. Thus, the “decision and responsibility”, in terms of ability to access and use contraceptives, rather than authoritative and powerful command, was up to girl within the relationship. On the other hand, girls are also confronted with individual and social norms concerning the idea of carrying condoms. Girls have to decide for themselves if they will take the responsibility for carrying condoms. In doing so, they can avoid risk-behaviour and lessen the male’s sense of duty. At the same time, girls have to face embarrassment when the initiative of carrying condoms comes from the female partner.

Although participants defined themselves as modern, in terms of knowledge and attitudes about contraceptives, most did not feel comfortable with the idea of girls carrying condoms. Stigma is attached to carrying condoms. Doing so connotes the woman/girl is of “loose character”; having “unnatural desire for men”; or “having sexual experience with many boys”. All of these are socially unacceptable behaviour for girls. Since women carrying condoms is socially taboo, this study raises the question to what extent girls can be seen as responsible for carrying and deciding to use contraceptives. Thus, women’s negotiation about contraceptives becomes a complex issue because they face conflicting ideas about personal risk, notions of equity, responsibility within the relationship as well as wider societal expectations.

As well, the embarrassment demonstrates how the concept of responsibility and choice of contraceptives is a fluid phenomenon. Modern and traditional contraceptives are perceived as a female domain bounded by notions of feminine roles and responsibility. In the Mozambican cultural setting, indigenous contraceptives are seen as a matter females share with *Massungukates.* These are old women who are venerated within the family and community for their expertise and wisdom in terms of advising women in matters related to SRH including marriage.

Thus, since sexual matters discussed among the women are viewed as secret, most responsibilities about modern and indigenous contraceptives still fall upon them. The notion of *Massungukates* – sharing knowledge with young people illustrates the importance of community and culture in understanding contraceptive use. However, it also illustrates how the concept of “stock of knowledge” is used to intuit, to obtain and manage pertinent information about contraception and sexuality. And, it demonstrates who is to be trusted, and what kind of behaviour is considered appropriate in a given social context. Advice related to safe sex of the people involved in discussing SRH can be a step forward for strengthening and supporting health behaviour and programmes designed for young people. In the next section, we discuss contraception practices.

### “It is challenging…oh, nobody likes it”! [contraceptive methods]: contraceptive practices

[Alice, 20 years-old, IDI, Boane headquarters].

Alice’s comment illustrates inconsistent, ambivalent and contradictory attitudes towards contraception among participants. In some cases, especially young adults, modern contraceptive prescriptions are abandoned in favour of natural and indigenous methods. This is typically done without consulting healthcare nurses. Meanwhile, ambivalence was more identified among adolescents who fail to take any decision concerning contraceptives.

Participants who had never been pregnant did not perceive ambivalence or contradictory and inconsistent birth control practices as risk behaviour. Contradictory practices were more predominant among young adults whose behaviour revealed a gap between what they knew and how they acted. The following extracts are illustrative of behaving in risky ways:

*Researcher: Did you use any contraceptive methods between your first and second pregnancy?*

*Hugy: Oh… I did nothing.*

*Researcher: At the moment, what do you do to prevent another pregnancy?*

*Hugy: I do nothing*…*it doesn’t depend only on me.* [Hugy, 17-year-old, FGD, Mahubo].

*You get crazy… you know… at that moment you are exited you forget everything… Forget to prevent pregnancy and also that you can be in a fertile period. In our age we want to try-out everything. If you do not try it; I mean follow what other adolescents do, or what we hear from our elder sisters, they will label you as old-fashioned. We are curious to know what sex is about.* [Joana, 18 years-old, FGD, Ndlavela].

*If the boyfriend does not want to use a condom, is because man tends to forget… I do it (risk-taking) and I think nothing will happen (exactly on that day).* [Marta, 21 years-old, FGD, Mahubo].

Our findings revealed most participants were conscious of their risk practices. Engaging in risk-taking was given as a way to avoid being labelled “old-fashioned”. Inconsistent, ambivalent and contradictory attitudes among young people are linked to individual choices; the self-concept of risk, and the pleasure having unprotected sexual intercourse. Also influencing the situation are power differences between the sexes, and previous experience of escaping the consequences of risk-behaviour. Most participants who had never been pregnant did not perceive ambivalence, contradictory or inconsistent birth control practices as risk behaviour.

When we asked participants what safe sex means, various answers were given. These included condom use, non-penetrative sex, and knowing the partner’s HIV status. However, trusting a partner’s sexual history, having sex with someone who looks healthy, casually knowing the partner but not bothering to have a medical check-up for STIs were all too common.

These mixed perceptions and practices reveal that when it comes to putting the concept of safe sex into practice, young people encounter many issues. Greater awareness about the need for both genders to carry and use condoms must be created, despite it being socially and culturally repugnant. The following section shows at which point some adolescents and young adults became conscious of the consequences of inconsistent use of contraceptives.

### “It was from that moment she became conscious”: attitudes towards contraception

[Ntilo, 17 years-old, FGD, Boane headquarters].

Consistent attitudes towards contraceptive methods were found largely among young adults who had experienced an unplanned pregnancy. Female participants tended to shift their behaviour because of the responsibility they had looking after a child, interrupting school, or having an unsafe abortion. Although male participants do not face constraints related to interrupting school, they shift their behaviour due to other consequences. Male responsibility includes supporting his child (and possibly his girlfriend), taking on a part time job, or leaving school entirely to work full time. Following early fatherhood, responsible young men opt to consistently use condoms or make sure his partner is using a reliable contraceptive method. As it follows:

*I also changed my mind because I had to help with emotional and costs for the baby… Actually I use condoms without interruption….or I remind her about the use of contraceptive. When she forget we use condom.* [Ntilo, 17 years-old, FGD, Boane headquarters].

*I suggest her to remove it* (to have an abortion)*. She started to become conscious… because she had acquired the notion of consequences.* [Kensany, 19 years-old, IDI, Ndlavela]*.*

*I have a child that I had before I started to plan. On that moment I was so much younger… but now I know that I am not prepared to have another child. That’s why I have to do family planning. In the past I had to drop out from the school.* [Wanga, 22 years-old, FGD, Mahubo].

Of the 12 participants (eight girls and four boys) who became early parents, most were in relationships of less than 2 years. An important issue raised by their experiences is the extent to which early parenthood acted as a causal factor increasing risk of economic and social disadvantages. However, these disadvantages were more common among girls due to providing primary caregiving responsibility. Consistent use of contraceptives for most participants was not often a preventive practice. Instead, it was applied as a catalyst of the consequences of unwanted pregnancies and early parenthood. This study also identified a level of institutional awkwardness and socio-cultural barriers interfering with the use of contraceptives. This is discussed in the next section.

### Notions about masculinity and femininity concerning contraception

Findings reveal existent notions and attitudes of masculinity and femininity among adolescents and young adults. These ideas contribute to their usually transitory perceptions of risk and inconsistent use of contraceptives. This is particularly evident among young male adults. Answers during interviews ran the gamut – “being a man [means to be] able to impregnate”; “a woman is able to be fertile” or; “a partner is not partner if both use barrier methods”. In addition, many female interviewees believed “a woman can only take the relationship seriously if the man is able to give her a child”.

Acting as a real “man” or “woman” – consciously adopting risk behaviour – or proving masculine/feminine identities were also used to test fertility. To impregnate or becoming pregnant are a means for young adults to test their virility/productivity. To their way of thinking, this proof justifies risk-taking. Thus, the perception of risk is understood as being necessary to obtain evidence needed at a specific moment in their lives:

…(W)*oman will not be satisfied if a man does not ejaculate at least one time inside her…* [Jaime, 23 years-old, FGD, Boane headquarters].

*She had the idea that I am not able to impregnate her. She was willing to get pregnant as proof.* [Tembe, 19 years-old, IDI, Boane headquarters].

*(…) Man always wants to feel it (unsafe sex). … A girlfriend does not know you if you never ever tried sex without protection…* [Cossa, 17 years-old, FGD, Ndlavela].

Being able to bear a child not only provides a sense of pride in preserving kin; it is also significant in maintaining the parents’ identities of femininity and masculinity. These practices are shaped by socioeconomics and gendering of cultural norms related to transitioning into adulthood.

However, testing female and male virility is a multifaceted and contradictory practice. Young people attempt to answer a social imperative, particularly for those who come from poor background. Showing the ability to conceive a child fulfils a personal need. It establishes a social role for a girl/woman and may possibly lead to marriage.

Conversely, testing fertility means one must accept the socio-economic risks associated with early parenthood. Thus, the notions of masculinity and femininity concerning contraception are indications of the use of “stock of knowledge” as an impulsive decision based on habits and expectations from the community within which young people live.

### Misconceptions and fear of contraceptive side effects

From our data, we learned adolescents and young adults were not consistent in using contraceptives due to the fear modern contraceptives might render them infertile. The most contested side effects of modern contraceptives were blood blockage, aging, frigidity, reduction of sexual desire, complaints of pelvic pain and haemorrhaging.

*It happened once with me; all month menstruating without stopping. I stopped using it* [IUD]. [Elsa, 16 years-old, FGD, Mahubo].

*Injections also contribute to earlier aging.* [Aniceto, 17 years-old, IDI, Ndlavela]*.*

*I started using an IUD but my friends told me to remove it because I am young. They say if you grow up the IUD will also grow up…* [Ana, 22 years-old, IDI, Boane headquarters].

This last quote demonstrates outside the clinics, a great deal of misinformation is in circulation. The effect of blood blockage is largely associated with infertility or loss of “the meaning given to women”. These fears have to be understood within their social norms which place a high burden upon individuals who are childless, and are not having their menstruations during their reproductive age.

For a woman to be infertile means she will be culturally blamed for not contributing to the continuation of the family lineage. The consequences of being infertile can include isolation, domestic violence and dissatisfaction within the marriage. Thus, misconceptions and fear of contraceptive side effects has greatly contributed to users shifting from one contraceptive method to another. As well, many women believe modern birth control methods do not meet their needs. This often results in ambivalent and contradictory practices and attitudes towards contraceptives in general.

### Limited parental communication with adolescents and young adults

Delicate dialogue between adolescents/young adults and their parents/guardians regarding sexuality constitutes a barrier to discussing contraception. In Boane, participants stated several cultural values created nearly insurmountable barriers. Firstly, the subject is taboo in most families. Second, initiating such a conversation may lead to episodes of domestic violence. Thirdly, subtle communication in the family context is the only way to avoid generational conflicts.

*I tried… (conversation with his father)… My father gave me an answer that … well…I did not like to hear. I asked why we (adolescents) should use condom… He said… hey…you are “donkey”… do not talk about matters that are not allowed for your age.* [Muando, 17 years-old, FGD, Ndlavela].

*At almost 16 years old I got pregnant… I was not interested in following through with the injections. I did not know the reason why I was taking it… In the beginning my mother and I used to go together to healthcare centre. When my mother told me to go alone, well I gave up because I did not know the reason. I asked her but she did not tell me…I become pregnant.* [Quissanga, 21 years-old, FGD, Mahubo]*.*

From the two quotes, it is clear communication between parents/guardians and a young person is limited. This constraint can be attributed to taboos and social significance given to the initiation of such topics at family level. Talking about contraception with adults is implicitly interpreted as an indication of already being sexually active. Within the family unit, it is common to discourage the use of contraceptives because of widespread misinformation about infertility when regularly using contraceptives.

Limited dialogue about sexuality only serves to obstruct broaching such a topic at the family level. It also reinforces the idea young people should learn about contraception within their social networks. It reveals how unwanted pregnancies can be a result of imposed cultural backgrounds leaving little space to discuss sexuality between older and younger generations. In both study sites, young people’s communication with parents was shaped by gendered notions of responsibility attributed to female and male roles. Girls often approached their mothers and boys to their fathers to initiate a conversation about sexuality.

### Power asymmetry between adolescents and young adults – nurses

Power asymmetry in communication between adolescents/young adults and clinic nurses are also listed as another barrier to contraceptive use. Participants described communications with nurses as sub-standard, limited, and often unilateral. Moreover, participants complained about the use of overly technical language and the absence of healthcare workers more attuned to the needs of adolescents and young adults.

*When I arrived at healthcare centre, they just asked me which symbol (contraceptive types)…I said “I do not know because it was the first time”… Then, they chose pills for me and said come back if something wrong happens with you. They did not explain to me about other contraceptives* [Nstala, 21 years-old, IDI, Mahubo]*.*

*I said* [I wanted the] *injection, and the nurse asked me why did I choose it. I said because I don’t want to have a child for now. Suddenly, she said “there is a side effect to injections which I did not understand”.* [Nhelete, 20 years-old, FGD, Mahubo].

The two quotations above are representative of inadequate attention from medical staff. It demonstrates sensitivity to uncertainties adolescents may have. There is little doubt nurses and healthcare workers are overworked. However, not allowing patients the right of informed choice is counterproductive. One-sided communication consisting of only medical instructions, are bewildering and can scare-off those seeking clarity and help.

Inadequate attention can also be linked to unequal power relations and poor interaction between young people and nurses. Long waiting times at clinics, lack of privacy, and brief interaction during consultations were commonly reported. Lacking enough time to sufficiently address young people’s needs has had direct negative impact upon the use and acceptance of SRH services.

## Discussion

This study reveals how policy initiatives for reducing sexual risk-taking and promoting contraceptive use can easily be overturned by inadequate attention. Little consequence is given to the plurality of notions held by adolescents and young adults. As well, social and medical barriers are equally disregarded. These barriers include ideas about indigenous contraceptives, restricted sexuality dialogue between adolescents/young adults and their parents/guardians. Also needing to be dealt with are: notions of masculinity and femininity surrounding contraceptives; misconceptions and fear of contraceptive side effects; imposed contraceptive choice; over use of technical language; and the absence of healthcare workers more attuned to the needs of adolescents/young adults. All of these influences produce negative outcomes. This is consistent with other studies revealing that insufficient attention to medical and social barriers leads to declining quality of health among adolescents and young adults [56–58].

In Ndlavela, there was a slight trend toward contraceptive acceptance by adolescents and young adults when compared to Boane. These variations can be associated to socio-cultural background, differences in knowledge as well as geographical location. Boane is located in rural Mozambique where the average fertility rate is 6.6 children per woman and the use of modern contraceptives is just 7 %. Its inhabitants are more deeply influenced by traditional, social and religious norms and the usual marital age for girls is 12 years [11, 23, 24].

Despite cursory differences between Boane and Ndlavela and the dissimilarity among participants from each study site, this study reveals a plurality of notions about contraceptives. A wide range of “stock of knowledge” has evolved and is often the basis for decision-making about the use and choice of contraceptives. Therefore, distorted information is believed and internalised in many different ways. This is consistent with other studies which indicate incomplete and distorted knowledge about contraceptives and risk-taking among sexually active adolescents [59, 60]. There are many different perceptions about what participants know and how they behave. As well, divergences concerning topics discussed during our conversations with participants often contradicted policymakers’ expectations. Consequently, they negated programmes aimed at improving young people’s health [8, 9, 61]. There is clear evidence of practical information being provided through existing services related to SRH. However, consistent contraceptive use and safe sex are complex and multidimensional practises. They not only involve contraceptive decision-making, but also the skills to negotiate reconsideration of social norms and gender relations. Young people have selected hierarchised and perceived risk in several ways according to specific contexts with which they were confronted.

In Ndlavela and Boane, boys’ conversations and participation differed from those of the girls by being peripheral and timid. When it comes to fertility control matters, women and men rarely engage in direct cross-gender conversation. This is because of the prevailing gendered social boundaries and social distances of their culture [62]. Although girls showed high level of awareness, they possess lower agency when it comes to making decisions about contraceptives and condom use. As well, there were differences about who participants felt should be responsible for contraceptive use and who actually was responsible. Likewise, some studies stress that even though adolescents indicated contraceptive responsibility should be shared by both partners, in practice this obligation mostly often fell upon the female [63, 64].

The notion of responsibility and decision making about contraceptives were also found to be complex. In Lowe’s study [65], where girls’ negotiation and control over contraception involved conflicting ideas about embodied risks, notions of equity within their relationship, and wider societal expectations. Men’s power over contraception seemed to lie in unspoken assumptions, with women often complicit in accepting them. Although women assert they have most control over contraception it was clear that many women were aware about their partner’s preference and decisions, and often made their decisions based on this. Most women felt conflict between their right to bodily autonomy and the expectations of equitable coupledom. Further, this study highlighted that girls felt an embodied sense of responsibility to prevent an unwanted pregnancy where boys appeared as an absent presence within girl’s contraceptive decisions [65].

These gender differences can and do lead to misunderstandings and miscommunications between partners. As well, it may influence individuals’ perceptions of reproductive, contraceptive matters, preferences and decisions. Thus, sex education programmes must address the range of concerns motivating young people’s engagement with contraception, safe sex and consistent use of contraceptives, because in many cases, it may not be a matter of choice. It is also important to consider position within the relationship and how this may impact on a woman’s ability to negotiate contraceptive use.

This study has also identified at what specific moment participants adopt roles/identities of masculinities and femininities, particularly among young adults. Both notions clarify the self-concept of risk behaviour. However, men and women do not act because of their role identities or psychological traits. Instead, they are influenced by concepts of femininity and masculinity adopted from their culture [66]. This meaningful expectation, as well as the significant role of parents in the mitigation of youth risk-taking was also found in other research. Amongst poorer social segments, other researchers have also concluded parenthood is seen as a sign of social status [67–69]. Motherhood is an esteemed state, particularly to young people coming from a traditional and less urbanised background. Such attitudes demonstrate the deeply ambivalent sentiments regarding the significance of maternity. However, it also reveals the need to answer a social imperative despite having to deal with the socioeconomic consequences of early parenthood [69]. By consistently using contraceptives, participants exhibited they were complying with the official discourse mandating pregnancy should be delayed to avoid socioeconomic consequences [8–10]. At the same time, by having the power to interrupt contraceptive use, they could also respond to the imperative to test their fertility.

Withdrawal, injections, pills, condoms, indigenous, and IUDs – in preferential order – were the favoured contraceptive methods. The IUD was the most contested due to beliefs associated with it. These included IUDs cause infertility, bleeding, aging, frigidity, reduction of sexual desire and pelvic pain. Similarities were also found in other studies showing a lower use of the IUD [70].

The prevalence of indigenous practices among young people, were likewise found in Agadjanian’s study, [71] illustrating the complexity and ambiguity of the notions of tradition – indigenous contraceptives; and innovation – modern contraceptives. Further, this author advises that in cases of opposition of formal/modern and informal/indigenous medicines, the latter surfaces and expands when and where the former fails to meet clients’ expectations.

Finally, outcomes from this study question fundamental principles underpinning health discourses concerning young people and the general sexual health of Mozambique. Mozambican public health discourses [8–10], as well as those from other countries [72, 73] emphasise the need for women to have self-esteem. However, they fail to acknowledge the dangers for women trying to affirm their female identity by adopting assertive behaviour to enhance their self-confidence.

This study has found dominant cultural ideas of female sexuality and power relations make it difficult for girls to negotiate safe sexual intercourse. If unequal power relations and cultural values are not taken into account, such government initiatives are likely to be unsuccessful in their attempt to improve the status of girls and young women. For instance, the stigma attached to girls carrying condoms implies they are sexually experienced and desire boys. Socially, this is utterly objectionable. In fact, the risk of losing one’s good reputation has been found to be more important than the risk associated with unwanted pregnancy and STIs [72, 74].

Lastly, differences in terms of using “stock of knowledge” to make decisions about safer sexual behaviour by young people in both study sites and among participants themselves in the same setting may provide support for programs or theories about safer sexual behaviour. This may be carried out by identifying challenges and strengths of rational and spontaneous decisions based on habit to build bridges between knowledge, practices and adolescent SRH policy to decrease health disparities and improve adolescent health. Through understanding of the “stock of knowledge” this study identified barriers and challenges concerning contraception and risk taking. Barriers and challenges when disclosed and addressed can lead to building programs more attuned to young people health needs and increase their involvement into SRH programs. Adolescents who rationally used the “stock of knowledge”; all those who used contraceptive consistently, showed a high level of awareness, and used condoms not only as barrier contraceptive but also as a practice to avoid STIs can be involved in programs/activities. Their input can be used to combine their knowledge and social action to achieve social change and improve the health of adolescents.

Nonetheless, this small-scale qualitative study has been hampered by some methodological limitations. There were certainly disadvantages associated with FGDs conducted with several people at one time [48]. However, all participants expressed themselves adequately since we had developed an effective way to restrain more talkative participants and encourage the less forward to speak up. Finally, due to availability of participants, existent taboos associated with the topic of sexuality, and costs related to the field work, FGDs were held with same gender groups of adolescents and young adults. Thus, the findings of this study have to be interpreted with caution and cannot be generalised.

## Conclusions

Exploring Mozambican adolescents and young adults’ experiences with contraception in light of the social context, reveals a plurality of notions about contraceptive methods. This has led to changing perceptions of risk-taking as well as inconsistent and ambivalent practices concerning contraception. Discrepancies found between knowledge and actual practice, are influenced by medical and sociocultural barriers.

Both girls and boys voiced the same concerns in terms of perceptibility, confidentiality, necessity for accurate information, and staff trained to better deal with their needs. However, if we are to dare/risk talking about the existence of “decision-making” concerning contraceptives and safe sex, it must be acknowledged that notions and practices of masculinity and femininity identities are culturally embedded. Typically, these are used to respond to social imperatives linked to parenthood as well as the stigma attached to carrying condoms.

Attitudes towards responsibility for contraceptive use and risk-taking are strongly gendered. These differences became critical, particularly for girls who are pushed into a position of responsibility for contraceptive use. In this social context, skills of negotiation and safe sex practices are complex and fluid issues. Imbalances of power within relationships mean it is difficult for women to continuously engage in safe sex or consistently use contraceptives.

In order to improve young people’s health, there is a need to: 1) pay attention to medical barriers – better training of health workers and recruitment of healthcare workers more attuned to the age group’s needs, 2) disseminating accurate information about IUD usage, 3) encourage male participation in contraception, 4) disseminate information about the disadvantages of early parenthood, including activities for girls’ empowerment within young people’s programmes, and 5) strengthening community programmes involving adolescents/young adults, parents/guardians, and health workers. Ultimately, a more holistic approach recognising all perceived risks, promoting a range of safe sex practices and situating safe sex within the context of these young people’s lives, is urgently required.

## Abbreviations

FGDs, focus groups discussions; HIV/AIDs, human immunodeficiency virus/acquired immunodeficiency syndrome; IC, informal conversations; IDIs, in-depth interviews; SRH, sexual and reproductive health; SSA, Sub- Saharan Africa; STIs, sexually transmitted infections

## Appendix 1

Interview guide.

**Table 4:** Data collection tool 1: In-depth interviews with clients of family planning aged (15–24)

Semi-structured interviews with young people aged 15-24	
**▪ Objectives: identify the knowledge, attitudes and practices about contraception and safe sex on which adolescent and young adults internalise and follow.**	
1. **Knowledge and attitudes related to contraception** 2. Have you heard about contraception/ and safe sex? Where? 3. What kinds of contraception do you know about? 4. Where do you go when you need contraceptives and information about SRH? 5. Who decides what type of contraception is to be used – you, your partner, providers or others? 6. What is your source of information about contraception and family planning? 7. What does safe sex mean to you? 8. What is your idea of risky sexual practice regarding contraception; and what does safer sex mean? 9. Who do you think has more control over contraception choice and use – you or your partner? **10.** What do you think about early parenthood and its consequences?	
A. **Identification of the practices related to contraception and safe sex.** 1. Have you ever participated on counselling services concerning contraception? 2. What do you do to prevent a pregnancy? 3. In what situations do you use or not use contraceptives? 4. What kind of contraception do you use? 5. Have you ever stopped using contraceptives? 6. Have you ever been pregnant? If so, was it intended or unintended?	
1. **The relationship between young people and health workers and parents or guardians.** 2. What do health workers say about contraception? 3. What is your relationship like with heath workers? 4. Do you feel comfortable with the information conveyed by providers? **5.** Are the SRH services taking into account your specific needs?	

**Estimated time**: 1 h – 1 h, 30 min

## Appendix 2

**Table 5:** Data collection tool 2: focus groups with users aged 15–24

Questions/activities for the participants: young people aged 15–24 years	
1. Have you ever seen or heard about these devices or pills?	
2. What do you know about contraception?	
3. When do you use or not use a contraceptive method?	
4. What do you do to prevent a pregnancy? Have you ever been pregnant?	
5. Where do you go for information about contraception?	
6. With whom do you talk about contraception and sexuality?	
7. Have you ever stopped using contraceptives?	
8. Have you ever participated in counselling sessions concerning contraception and SRH? If so, where?	
9. What does safe sex mean?	
10. What is your idea of risky sexual practice regarding contraception; and what does safer sex mean?	
11. Who do you think has more control over contraception choice and use – you or your partner?	
12. Who decides what type of contraceptive method is to be used?	
13. Who takes on the responsibility of contraception?	
14. What do you think about early parenthood and its consequences?	
15. Describe the relationship between you and healthcare providers when discussing or attending SRH services? And with your parents?	
16. What kind of contraceptives do you currently use? (Question to be asked in private.)	

**Estimated time**: 1 h- 1.30 min to 2 h
